# The ethical justification for inclusion of neonates in pragmatic randomized clinical trials for emergency newborn care

**DOI:** 10.1186/s12887-019-1600-x

**Published:** 2019-07-02

**Authors:** Dan Kabonge Kaye

**Affiliations:** 1 College of Health Sciences, Department of Obstetrics and Gynecology, Makerere University, P.O. Box 7072, Kampala, Uganda; 2 Berman Institute of Bioethics, Johns Hopkins University, 1809 Ashland Avenue, Baltimore, 21205 USA

**Keywords:** Neonates, Vulnerable populations, Vulnerability, Randomized clinical trials, Pragmatic randomized clinical trials

## Abstract

**Background:**

Research guidelines generally recognize vulnerable populations to include neonates with the aim of enhancing protections from harm. In practice, such guidance results in limiting participation in randomized clinical trials (RCTs). Yet while medical care of neonates should be based on best research evidence to ensure that safe, efficacious treatment or procedures are used, this seldom happens in contemporary practice.

**Discussion:**

The compelling need to generate information on effectiveness and safety of procedures and medications that are already in use during neonatal care has led to increase in calls for pragmatic randomized clinical trials (PCTs). This raises ethical concerns as to whether exclusion of the vulnerable populations from research participations constitutes harm. First, neonates are denied access to both potentially beneficial research outputs and an opportunity to generate data on how interventions or medications perform in diverse clinical settings and inform clinical decision-making. Secondly, risks and harms in PCTs may differ from traditional RCTs, and can be reduced by modifications in study designs. The latter may involve assessment of effectiveness of comparable medication, devices or practices (whose safety data is available), randomization at the group level rather than at the individual level, avoidance of invasive and innovative study procedures, reliance on locally available data on relevant patient outcomes, and employment of procedures that tend to meet the criteria of minimal risk for human subject research. Thirdly, informed consent procedures should be modified from those of traditional RCTs, as neonates in traditional RCTs may be vulnerable to different extents in PCTs. Lastly, regulatory and oversight procedures designed for traditional RCT settings need modification, as they may not be translatable, feasible, appropriate or even ethical to apply in PCTs.

**Conclusion:**

The principle of justice, commonly interpreted as preventing an inequitable burden of research, should also allow fair access to potential benefits from PCTs for neonates and other vulnerable populations. Under certain conditions, prospective randomized trials involving neonates should be ethically permissible to allow inclusion of neonates in research. This may require modification of the research design, consent procedures or regulations for research oversight.

## Background

While traditionally, protecting vulnerable research participants from harm meant excluding them from research, this exclusion may be harmful, and yet neonatal clinical research is hard to perform, is very expensive, and may generate unique ethical concerns [1, 2]. Whereas medical care of critically ill neonates should be based on best research evidence to ensure safe, efficacious treatment or procedures are used, this seldom happens in contemporary practice [1–3]. Even innovative therapy from newly introduced treatment or modifications to an existing therapy with unproven efficacy and side-effect profile, are widely used in the best interests of a patient [3]. The need for innovation is driven by the obligation to rescue, often in emergency circumstances that do not permit prolonged deliberation [3, 4]. Traditionally, protecting vulnerable research participants from harm practically meant excluding them from research. However, this exclusion may be harmful [1–4]. This creates conflict between the need to protect the vulnerable neonates and the obligation to protect them from routinely-used potentially ineffective medications or interventions, which are ethically acceptable because they offer hope in the face of an otherwise bleak outcome [5].

Parental consent for the participation of their neonate in neonatal research is influenced by the quality of the information delivered and the interaction between parents and investigators [6]. This calls for a need to provide detailed information in a way that makes it possible for parents to understand and make informed decisions. This similarly applies to pragmatic randomized clinical trials (PCTs), which are studies that seek to optimize care and ensure rational, safe, equitable and timely allocation of scarce neonatal critical care resources on medications or procedures that are already in use through pragmatic clinical trials [5]. PCTs are defined as trials that seek to compare relevant alternative clinical practices for which there is known practice variation among a diverse population of study participants, recruiting participants from heterogeneous usual practice settings, and which collect data on a variety of health outcomes [7]. The goal of designing such clinical trials is to streamline and simplify trial design in order to answer questions that inform decision-making about health and healthcare [8].

Features commonly associated with PCTs may raise ethical concerns [8, 9]. Such features include assessment of effectiveness of comparable medication, devices or practices whose safety data is available (to some extent), randomization at the group level rather than at the individual level, reliance locally available data on relevant patient outcomes, and employment of procedures that tend to meet the criteria of minimal risk for human subject research [8, 9].

Research on neonates is scientifically and ethically necessary to establish the efficacy and safety of drugs widely used in neonatal care, but must be carefully designed to balance potential risks and benefits [1–3]. A study is of minimal risk when the probability of harm is low and/or the harms that might accrue are satisfactorily minor [10]. The inherent riskiness of interventions should not be considered as risks of the research if these same procedures would necessarily be conducted as part of routine clinical care for eligible participants patients [10]. I present an argument, from a consequentialist standpoint, that research with neonates may be permissible where potential benefits for future patients (social value) outweighs the risks to individual participants, the risk level notwithstanding, and as long as there are appropriate modification of the study design, consent process and study procedure to reduce potential harms. This is the ethical way of addressing knowledge and research gaps regarding drug use during neonatal care.

## Main text

### The ethical argument for inclusion of neonates in research

The drive for PCTs is embedded in the desire to assess whether drugs routinely used for similar disorders are comparable in terms of both safety and effectiveness, thus more or less meeting the criteria of minimal risk research [7–9]. There is risk and potential harm in using medications or devices of doubtful efficacy and safety during obstetric or neonatal care. While pregnant women fall sick, and many women conceive while undergoing medication for chronic illness, there is limited data on safety of many medications women take during pregnancy [11, 12] . Likewise, there limited data on some medications, devices or procedures used during the neonatal period. There is a concept of minimal risk acceptable in research on vulnerable populations like neonates [13, 14]. There is need to rethink vulnerability and required safeguards by employing approaches that modify the research design, informed consent process, data collection procedures, study interventions and research regulation to support the inclusion of vulnerable subjects while preserving their safety, welfare and interests [14].

Often, the regulations may seem to offer over-protection, as they permit research on vulnerable populations only if they pose no more than minimal risk [15–19]. *Minimal risk* is defined as, ‘the probability and magnitude of harm or discomfort anticipated... are not greater... than those ordinarily encountered in daily life or during the performance of routine physical or psychological examinations or tests’ [15–19]. For minimal risk PCTs, the additional protections afforded vulnerable subjects, primarily in the informed consent process, may be unnecessary or overly complicated [8, 9], thus rendering the safeguards from current regulations and institutional practices inappropriate, infeasible or unethical [8, 9, 14, 20]. Besides, there is potential harm from exclusion of neonates from research participation. For instance, one effect of over-protection from research is evident in the contemporary standard practice of allowing administration of novel untested drugs in neonates and other pediatric populations, especially to those with critical illness, thereby exposing them to potentially ineffective or even harmful medications [21]. While research may not be ethically justifiable for potentially exposing the most vulnerable patients to high risk of harm with no potential for individual benefit, there is potential harm in using medications of unknown efficacy or safety in pregnant women and neonates [11, 14, 21, 22].

There is need to rethink vulnerability. Vulnerability ought not to be viewed as intrinsic to a specific population but as situational, where the individual participant, study characteristics and circumstances intersect [22]. Table 1 identifies seven vulnerability characteristics that can be extended to all vulnerable populations. Individuals with a critical illness may have situational vulnerability “in which medical exigency prevents the education and deliberation needed to decide whether to participate in the study” [22]. Thus, individuals are not inherently vulnerable simply due to membership in a group; but characteristics of the individual in the context of the study are the determinants of vulnerability and the need for extra protection [22]. Thus, as Table 1 shows, multiple vulnerabilities may co-exist. Vulnerability in sick neonates who require emergency care ought to be viewed in terms of the individual attributes and characteristics of the subjects in the study, which include being neonates, being unable to provide consent, being in a position where communicating to the parents may not be easy for them to understand the disclosed information, and being at risk of being provided with medication or practices of unproven safety and effectiveness.

**Table 1:** Individual characteristics of vulnerability to consider by population

Population	^a^Situational	^b^Incapacitational	^c^Juridic	^d^Deferential	^e^Social	^f^Medical	^g^Allocational
Children	X	X	X	X	X	X	X
Disadvantaged persons	X			X	X		X
Human fetuses	X						
Neonates	X	X					
Persons with mental/physical disability	X	X	X	X	X	X	X
Pregnant women	X			X			
Prisoners	X		X	X	X		X
Ethnic or racial minorities	X			X	X		
Critically ill patients	X	X		X	X	X	

^a^“In a situation in which medical exigency prevents the education and deliberation needed to decide whether to participate in the study.” For instance, newborns with neonatal emergency conditions

^b^Lacks “the capacity to deliberate about and decide whether to participate in the study.” For instance, parents of newborns may not have enough time for deliberations on whether to participate

^c^“Liable to the authority of others who may have an independent interest in that participation”

Often, conflicts of interest in the investigators may influence participation

^d^“Given to patterns of deferential behavior that may mask an underlying unwillingness to participate”

^e^Belongs “to a group whose rights and interests have been socially disvalued”

^f^Has “been selected, in part, because of the presence of a serious health-related condition for which there are no satisfactory remedies.” This is situation common in severe neonatal emergencies where viability is doubtful (need to rescue)

^g^“Lacking in subjectively important social goods that will be provided as a consequence of participation in [the] research”

Adapted from Reference [22]

There is need to balance obligation for both clinical research and care, as research in pragmatic RCTs is embedded in clinical care [7–9]. Despite being closely related, clinical research and medical care are often separated by clear boundaries, especially in traditional RCTs [23]. Regarding purpose, clinical research is conducted to generate generalizable knowledge useful for future patients, whereas clinical care aims to promote the well-being of individual patients. The difference in purpose raises the distinction between therapeutic (possibility of cure) and non-therapeutic research (research on biological or physiological mechanisms) as indicated in the Declaration of Helsinki [24], which distinguishes between therapeutic and non-therapeutic scientific research. Research in which the subject may personally benefit from the intervention is considered therapeutic, for instance when two approved therapies are compared as in the case of PCTs. If informed consent is deemed appropriate but a person is unable to communicate, consent may be waived or a proxy can give consent (in the interest of the patient) [24]. A study is regarded as non-therapeutic if no health-related direct benefit for a specific patient is anticipated, for example when physiological processes are evaluated. However, the progress towards patient-oriented research and patient-centered medicine blurs the boundaries between clinical research and care [25]. Besides, the widely-accepted need for gradual standardization of medical care and innovations in care contribute to not only closer ties between clinical research and medical practice, but also integration of both activities [25]. This however, requires addressing important ethical and methodological challenges.

### Protections and safeguards for neonatal RCTs

Research guidelines operationalize the above ethical principles. The Belmont Report outlines three fundamental ethical principles for human subjects research: respect for persons, beneficence, and justice [23]. Protections and safeguards built into research to prevent harm to individuals or groups have underlying principles: 1) Respect for persons (also called respect for autonomy) encompasses both the right of autonomous individuals to make free decisions about research participation consistent with their own values and preferences. This also implies the right of vulnerable persons to be protected from research risk or medical care. 2) Justice or equity necessitates a fair distribution of the benefits and burdens of research, and is often considered at both the individual and the population level. While commonly interpreted as preventing an inequitable burden of research to vulnerable populations [23], justice should also prevent inequitable access to potential benefits from PCTs for neonates and other vulnerable populations (that may follow exclusion from research participation). Thus, the principle of justice is an important rationale for policies promoting inclusion in research. 3) Beneficence is the obligation to do good and to avoid harm, by conducting research that has social value and potential to promote well-being, and avoidance of risks and harms. In research, one commonly weighs risks and benefits to ensure a favorable benefit-risk ratio.

These principles serve as the foundation for federal research regulations, which are codified as 21 CFR 50 (FDA regulations on the protection of human subjects) and 45 CFR 46 (the Common Rule) [15–19]. Informed consent guidelines follow from the principle of respect for persons. In practice, informed consent entails providing information (including purpose, rationale, benefits, potential risks and alternatives to participation), assessing comprehension of the information provided, and ensuring the consent is voluntary and not coerced by circumstances or persons involved [23]. While these policies establish important protections, they view clinical research as a highly controlled system distinct from medical practice, a divide that results in failure to provide decision-makers with high-quality evidence to make the best choices in medical practice, even through PCTs [7, 26, 27].

### Implications of modifications in study designs, consent process and implementation approach for ethical regulation in neonatal PCTs

Appropriate PCTs for neonatal clinical research may have diverse interventions (medical, behavioral, and/or technological) targeting diverse participants (neonates, clinicians, and/or healthcare system processes), and multiple overlapping intervention types and targets may co-exist within a single trial [28], with some involved more directly than others. This diversity in design influences the regulatory and ethical considerations for these trials. Modifications in the consent process in neonatal PCTs include waiver of consent, deferred consent, opt-in and opt-out procedures, and integrated consent [20, 29, 30]. The motivation to protect patient-subjects from potential harm through informed consent is primarily rooted in concerns over introduction of new and often unknown risks associated with experimental interventions [10, 28]. There is also possibility that researchers have divided loyalty to individual research participants (driven significantly by the desire to produce generalizable knowledge for the benefit of others), another problem of the research-care distinction [28–30].

Waiver or other modification of informed consent may be done if the study is of sufficiently low risk [10, 20]. Waiver of consent should be limited to cases in which the risks of participation are low and where more engaged consent options are infeasible [15–20]. If a study using a cluster-randomized design were approved with a waiver of the requirement for consent, then a study of the same interventions that employed randomization at the level of the individual should also qualify for similar modification of consent [10]. A minimally arduous consent process should be appropriate for minimal-risk studies, and could be more effective in helping prospective research participants focus on the most important elements of a study, rather than on the rituals of a “full” informed consent process [31]. Transparency can solve some of the dilemma’s surrounding randomization and informed consent [31]. Thus there is need for investigators to exhibit more transparency regarding the informed consent procedures used in neonatal research. While informing potential participants about the aims and procedures of a trial when seeking their consent is usually reported, there are usually no details about the nature of information disclosed, the methods used to disclose the information, or procedures used to assess comprehension. The exact information that is given to potential participants may often not be understood by them. Details about informed consent procedures of PCTs for neonatal research should be reported transparently with the essential features of the information for participants summarized to inform more understanding of the consent procedures [32].

There is need to modify the requirements for oversight. Institutional Review Boards (IRBs) have the responsibility to determine if the proposed study has value, highlights clinical equipoise and addresses an important question related to variations in clinical practice [10]. Clinical equipoise refers to the degree of practice variation within a community of practitioners, who don’t seem to agree whether one medication or practice is superior to others in the same situation or similar condition. The degree of consensus among the clinical community about the best treatment corresponds to a lesser or greater degree of justification for conducting a PCT [10]. This approach offers a method of assessing the likelihood of a participant missing out on standard care, that is, that a participant in a research study would receive a different treatment than that person would have otherwise received as part of routine practice [7–10, 33]. Where there is widespread practice variation, the resulting allocation of treatments approximates the allocation that will result from randomization [33].

IRBs also have obligation to assess the level of risk involved in the study [10]. RCTs in neonates may undergo expedited review by IRBs, in contrast with studies judged to entail more risk, and the minimal risk threshold is a prerequisite for performing certain types of studies in neonates [15–19]. While traditional RCTs study new therapies in highly controlled settings, PCTs assess individual or group outcomes of therapies in ongoing use, in heterogeneous populations in real-world clinical settings [7–9]. They commonly evaluate interventions using in routine practice, assess outcomes in usual care settings, and rely on routinely collected data [7–9]. There are, however, practical challenges in the application of ethical principles and regulations to PCTs in vulnerable populations [7–9, 33–38]. Multiple study designs may be used to answer pragmatic research questions in neonatal research (ranging from observational studies and RCTs to mixed methods research and implementation research) with each design requiring different types and degrees of engagement with participants in order to successfully test interventions and assess necessary data on the outcomes [39, 40].

In assessment of risk, IRBs ought to have a pragmatic approach where risk is assessed on a continuous scale rather than a dichotomy [28]. To adequately assess the net risk of complex PCTs involving multiple types of interventions and target groups, IRBs ought to ensure that potential harms and benefits for each type of participant are considered carefully [28]. The risks to each participant should be reasonable in relation to the possible benefit [10]. Likewise, IRBs should evaluate risks to others (non-participants) who may be affected by the clinical trial [41–43]. Currently, most IRBs assess levels of risk as a dichotomy of minimal risk and greater than minimal risk, whereby studies are either minimal risk or greater than minimal risk. From a regulatory perspective, if all studies in the latter category are treated similarly, then any study that exceeds the minimal-risk threshold will undergo similar regulation to that applied to the riskiest studies [10, 41–43]. Considering that IRBs tend to be very cautious and risk-averse, there might be misallocation of regulatory resources to studies that do not merit such oversight [41, 42].

Studies that are very low risk but may not meet current criteria for minimal risk ought not to be treated like the riskiest studies [10]. A similar concept is employed in certain pediatric research (that offer no potential for direct benefit to participants and meet several other regulatory requirements) which include a category of research that entails “a minor increase over minimal risk” [10]. Conceptually, such a spectrum better reflects the realities of PCTs, where risks of research participation may be comparable to the risks of treatment outside the research protocol, but are best considered as a minor increase over minimal risk [10]. The consent process ought to focus on the differences in known and anticipated risks between treatments for those who are enrolled in the study compared to those not enrolled [43]. Such a conceptualization can be able to distinguish risks attributed to the disease, from those attributed to the treatments or those attributed to the research itself.

Therefore, IRBs should have clear, transparent protocols and guidelines indicating agreed-upon methods for determining the risk level of proposed neonatal research, and delineating consistent procedures by which IRB decisions are taken [41, 42]. From the Common Rule [15–19], IRBs are key in evaluating the net risks of PCTs, ensuring that risks to subjects are reasonable in relation to anticipated benefits, as well as the value of the knowledge that may reasonably be expected to result [28]. However, it is permissible that investigators conducting a study with a single protocol at multiple research sites that have separate IRBs may find differences in the decisions taken by the different IRBs related to review, procedures or consent process, as this variability may represent responsiveness to the local culture or values of the different institutions [10]. Since, in multicenter trials, such variability becomes problematic by creating different consent requirements at different sites, and potential selection biases in subject recruitment, there is need to identify a single IRB to be the IRB of record for the study [10, 34]. In evaluating risks and benefits, IRBs should consider only those risks and benefits that are consequences of research participation (as distinct from risks due to participants’ illness and risks and benefits of therapies subjects would receive even if not participating in the research) [28, 34, 36]. With this regard, IRBs should not consider possible long-range effects of applying knowledge gained in the research (for example, the possible effects of the research on public policy) as among those research risks that fall within their responsibility [28, 34].

Questions arise on the role of procedures such as randomization and blinding in neonatal PCTs on the risks and harms associated with PCTs. Risks can be relevant to decision-making even when they are minimal, and may be pertinent if they are diverse [28]. The risks and benefits created by random assignment to one of two or more treatments may differ from the expected risks and benefits of treatment selection by the patient and doctor based on weighing the available evidence [10, 28, 44]. In the setting of clinical equipoise, randomization per se does not increase risk or decrease benefits for participants in RCTs, compared with individualized physician-patient decision making based on clinical judgment [32, 44]. Different randomization protocols can be considered for which informed consent may not always be necessary: treatment options may be randomized according to time, population or place (health facility) [31]. In certain conditions, individual patients may even be randomized to different treatment options consecutively to serve as their own control [31]. This necessitates full stakeholder engagement and transparency, with emphasis on the need to embed research into healthcare processes, including that individuals may participate in a research protocol without prior information, provided the study poses no risk of harm secondary to participation [25, 27, 29, 31].

Questions also include what constitutes research versus a quality improvement under current regulatory guidelines [45], how the criteria for determining what is minimal risk research be appropriately applied [28, 42, 43], and whether alteration of informed consent process is ethical and justified [35, 41, 45, 46]. However, the distinction between clinical practice and research is increasingly blurred as healthcare system advances toward a learning healthcare system. The learning healthcare system addresses the challenge created by rigid application of guidelines on obtaining informed consent, consequently limiting both the number and representativeness of participants in a study [25]. This approach is in line with the obligation of (academic) medical centres to both provide optimal care and advance clinical expertise, as enshrined in Article 6 of the Declaration of Helsinki: ‘Even the best proven interventions must be evaluated continually through research for their safety, effectiveness, efficiency, accessibility and quality’ [24].

### Enhancing the role of gatekeepers to address challenges associated with neonatal participation in RCTs

Successful implementation of PCTs for neonatal research requires that investigators have access to resources such as financial support, institutional infrastructure (clinics, facilities, staff), eligible participants patients, and patient data) and support of the gatekeepers (people or entities with ability to allow or deny access to the resources required to support the conduct of clinical research) [47]. These include research sponsors, regulatory agencies, institutional review boards, health system and other organizational leadership, research team leadership, human research protections programs, advocacy and community groups, and clinicians [47]. The need to address the limited data on safety and efficacy of neonatal medication calls for a need to address challenges related to practical application of the regulatory and ethical paradigms through PCTs [8, 9]. This requires development of guidelines and regulations to nurture transparent decision-making processes as they engage the different stakeholders, as well as harmonization and streamlining of the research oversight process [34, 45, 46].

Gatekeepers (sponsors, regulatory bodies and IRBs) should be sensitized on the ethical tensions inherent in neonatal RCTs (including PCTs) related to concern for potential harms associated with neonatal research, as well as to concerns of individuals, groups, and communities affected by the gatekeepers’ decisions, (including need to balance protection from harm and maximization of benefits) [47]. Depending on the research design, the types and targets of PCT interventions and the outcomes, the assessment of the net potential risk (understood as the balance of potential harms and benefits) may vary in different studies [28, 29, 41]. As the balance between protection and access progressively shifts in favor of increasing access to pediatric research in general and neonatal research in particular, it becomes increasingly crucial to ensure the protection for these vulnerable participants [40, 41]. Responsibilities of gatekeepers also include stewardship of financial, human, and other organizational resources needed to ensure ethical inclusion of neonates in research [47]. The fundamental protection for research subjects, namely the informed consent of the parents or guardians before any recruitment, is not reasonable in true emergency situations common in neonatal research, and so modifications of the consent process are necessary [37, 41, 48].

Other regulatory procedures common to traditional RCTs may need to be modified to involve more stakeholder engagement and dialogue. Current oversight procedures in traditional RCTs may be inappropriate, restrictive, costly and time consuming [37, 38, 41, 48]. First, in PCTs, patients and clinicians may be called on to participate as designers, investigators, intermediaries, or subjects of PCTs [39, 40]. Besides, different members of the healthcare tea and the healthcare system itself may be affected directly or indirectly before, during, or after study [37, 39, 41]. Secondly, there is need to define obligations and responsibilities of investigators and the different stakeholders [40]. Health research regulations typically do not define obligations in relation to the individual characteristics of human subjects, with few exceptions for vulnerable populations [15–19]. Thirdly, while interventions in PCTs may pose more than minimal risk, they address questions that directly inform decision-making and quality improvement goals [40, 41]. However, there may be uncertainty regarding what specific risks ought to be considered as risks of research, rather than as the inherent risks of the neonate’s disease or the risks of the treatments that neonates receive even if not enrolled in a research project, so as to assess incremental risks of PCTs [10, 28, 41–43].

Concerns about protecting the privacy of participant data, though significant, must be balanced with the imperative to learn from the data gathered in routine clinical practice and through PCTs [25, 49]. Data anonymization (which is key in protecting privacy) is not suitable for PCTs [25, 49]. Regulations on privacy include, in the case of research using identifiable information, seeking the prior consent or authorization of the individual. With large difference between the ideal informed consent and the practical informed consent that characterizes PCTs and learning healthcare systems, such authorization is infeasible [49].

Lastly, there is a need to develop clinical trial designs and trial sequencing strategies that are tailored to neonates and to generate data to support rational drug-dosing guidelines in neonates, particularly to evaluate medications that are used off label neonatal disorders. One of the innovations is using adaptive trial designs, which promote flexibility through introduction of pre-specified modifications in the design or statistical procedures of an on-going trial depending on the data generated from the concerned trial [50].

Lastly, avoiding all potentially adverse exposures, including medications to improve health, may prevent some of the adverse effects of these medications on pregnancy outcomes. However, it is not always possible to avoid taking medications before or during pregnancy. Women who desire to conceive may experience short-term and long-term health conditions of variable severity that must be managed with medication. Besides, many women with ongoing treatment with such medications may have unintended pregnancy. In a retrospective study from eight health maintenance organizations, researchers estimated that approximately 59% of pregnant women were prescribed a medication other than a vitamin or mineral supplement at some time before or during pregnancy [51]. The use of over-the-counter non-prescription medications during pregnancy may be even higher, and many women take a dietary or herbal supplement other than multivitamins or folic acid while pregnant [52, 53]. Discontinuing medication for a serious condition when a woman becomes pregnant may have profound, long-term implications both for her health, her pregnancy and that of her baby [54]. Preconception care could provide the opportunity to optimize a woman’s use of medications in preparation for pregnancy. This may be achieved through identification of patterns of medication use before pregnancy occurs and making necessary adjustments to those patterns in order to avoid the use of nonessential medications, minimize exposure to medications known to be harmful to the embryo or fetus or pregnancy continuation [54]. Relevant adjustments of the dose, route of administration, and timing of essential treatments can optimize maternal health before conception and each stage of pregnancy while safeguarding the embryo, fetus, and infant [54].

## Conclusion

Under certain conditions, prospective randomized trials involving neonates should be ethically permissible to allow inclusion of neonates in research. This may require modification of the research design, consent procedures or regulations for research oversight, especially if the studies are designed as pragmatic randomized clinical trials or as part of a learning healthcare system. This approach will facilitate bridging gaps in the evidence on safety and effectiveness of medications that are currently being used for neonatal care, some of whose efficacy or safety is doubtful.

## Data Availability

Not applicable

## Abbreviations

IRB: Institutional Review Boards
PCT: Pragmatic clinical trials
RCT: Randomized clinical trials
